# Clinical Practice Variation and Outcomes for Stanford Type A Aortic Dissection Repair Surgery in Maryland: Report from a Statewide Quality Initiative

**DOI:** 10.1055/s-0040-1714121

**Published:** 2020-11-05

**Authors:** Michael Mazzeffi, Mehrdad Ghoreishi, Diane Alejo, Clifford E. Fonner, Kenichi Tanaka, James H. Abernathy, Glenn Whitman, Rawn Salenger, Jennifer Lawton, Niv Ad, James Brown, James Gammie, Bradley Taylor, On behalf of the Investigators for the Maryland Cardiac Surgery Quality Initiative

**Affiliations:** 1 Department of Anesthesiology, University of Maryland, Baltimore, Maryland; 2 Division of Cardiothoracic Surgery, Department of Surgery, University of Maryland, Baltimore, Maryland; 3 Division of Cardiac Surgery, Johns Hopkins University School of Medicine, Baltimore, Maryland; 4 Division of Cardiothoracic Surgery, Maryland Cardiac Surgery Quality Initiative Inc., Baltimore, Maryland; 5 Department of Anesthesiology, Johns Hopkins University, Baltimore, Maryland; 6 Department of Cardiothoracic surgery, St. Joseph Medical Center, Towson, Maryland; 7 Department of Cardiothoracic Surgery, Washington Adventist Hospital, Takoma Park, Maryland; 8 Department of Surgery, University of Maryland, Capital Region Health, Cheverly, Maryland

**Keywords:** aortic dissection, quality, cardiac surgery

## Abstract

**Background** Stanford Type A aortic dissection repair surgery is associated with high mortality and clinical practice remains variable among hospitals. Few studies have examined statewide practice variation.

**Methods** Patients who had Stanford Type A aortic dissection repair surgery in Maryland between July 1, 2014 and June 30, 2018 were identified using the Maryland Cardiac Surgery Quality Initiative (MCSQI) database. Patient demographics, comorbidities, surgery details, and outcomes were compared between hospitals. We also explored the impact of arterial cannulation site and brain protection technique on outcome.

**Results** A total of 233 patients were included from eight hospitals during the study period. Seventy-six percent of surgeries were done in two high-volume hospitals (≥10 cases per year), while the remaining 24% were done in low-volume hospitals. Operative mortality was 12.0% and varied between 0 and 25.0% depending on the hospital. Variables that differed significantly between hospitals included patient age, the percentage of patients in shock, left ventricular ejection fraction, creatinine level, arterial cannulation site, brain protection technique, tobacco use, and intraoperative blood transfusion. The percentage of patients who underwent aortic valve repair or replacement procedures differed significantly between hospitals ( *p* < 0.001), although the prevalence of moderate-to-severe aortic insufficiency was not significantly different ( *p* = 0.14). There were no significant differences in clinical outcomes including mortality, renal failure, stroke, or gastrointestinal complications between hospitals or based on arterial cannulation site (all *p* > 0.05). Patients who had aortic cross-clamping or endovascualr repair had more embolic strokes when compared with patients who had hypothermic circulatory arrest ( *p* = 0.03).

**Conclusion** There remains considerable practice variation in Stanford Type A aortic dissection repair surgery within Maryland including some modifiable factors such as intraoperative blood transfusion, arterial cannulation site, and brain protection technique. Continued efforts are needed within MCSQI and nationally to evaluate and employ the best practices for patients having acute aortic dissection repair surgery.

## Introduction

Stanford Type A aortic dissection is a life-threatening disease which is associated with a mortality rate of 30% by 48 hours in patients who receive only medical therapy. 1 Further, if surgery is not performed, mortality increases by 1 to 2% per hour. 2 Surgical repair is the gold standard for Stanford Type A aortic dissection. In one review of the Society for Thoracic Surgeons (STS) database, the operative mortality was 17% for 2,982 acute aortic dissections. 3 Interestingly, among 640 reporting hospitals in North America, the median number of cases performed per hospital was 3, suggesting that in many hospitals there is limited surgeon and institutional experience with operative management of these patients. 3

To date, few studies have examined statewide practice variation for Stanford Type A aortic dissection repair surgery. This type of analysis is important because many low-volume cardiac surgery hospitals perform Stanford Type A aortic dissection repair surgery and previous studies suggest that their outcomes may be worse. In one study of over 5,000 acute aortic dissection patients in the United States, there was a strong inverse relationship between hospital cardiac surgery volume and mortality. 4 In the same study, the operative mortality rate was 10% higher in hospitals that did less than one aortic dissection surgery per year on average during the 5-year study period, representing 22% of hospitals in the study. Mortality was particularly high in hospitals that had low overall cardiac surgery volume with hospitals in the lowest quartile having an approximately two-fold higher mortality than those in the highest quartile. 4

The purpose of our study was to explore practice variation and differences in outcome between cardiac surgery hospitals in Maryland that performed Stanford Type A aortic dissection repair surgery. Furthermore, we sought to explore differences in arterial cannulation site and brain protection techniques and how these factors impacted patient outcomes. We hypothesized that there would be significant practice variation between hospitals and that some practices (e.g., axillary cannulation) would be associated with superior outcomes.

## Materials and Methods

### Patients

Patients were identified using the Maryland Cardiac Surgery Quality Initiative (MCSQI) database. This database contains pooled Society of Thoracic Surgeons (STS) data for all cardiac surgery hospitals in Maryland. Adult patients having Stanford Type A aortic dissection surgery between July 1, 2014 and June 30, 2018 were identified in the database using the “aortic procedure performed” field (sequence no.: 2128) and “urgent or emergent reason” field (sequence no.: 1990; Fig. 1 ). Patients were included if their urgent or emergent reason was “aortic dissection.” Patients who had aortic dissection surgery related to iatrogenic dissection from aortic cannulation were excluded. Patients were also excluded if they were missing key study data such as arterial cannulation site and brain protection technique. The Institutional Review Board at the University of Maryland, Baltimore, exempted the study and gave a waiver of written informed consent, as the dataset was fully deidentified and it was not human subjects research.

**Fig. 1:**
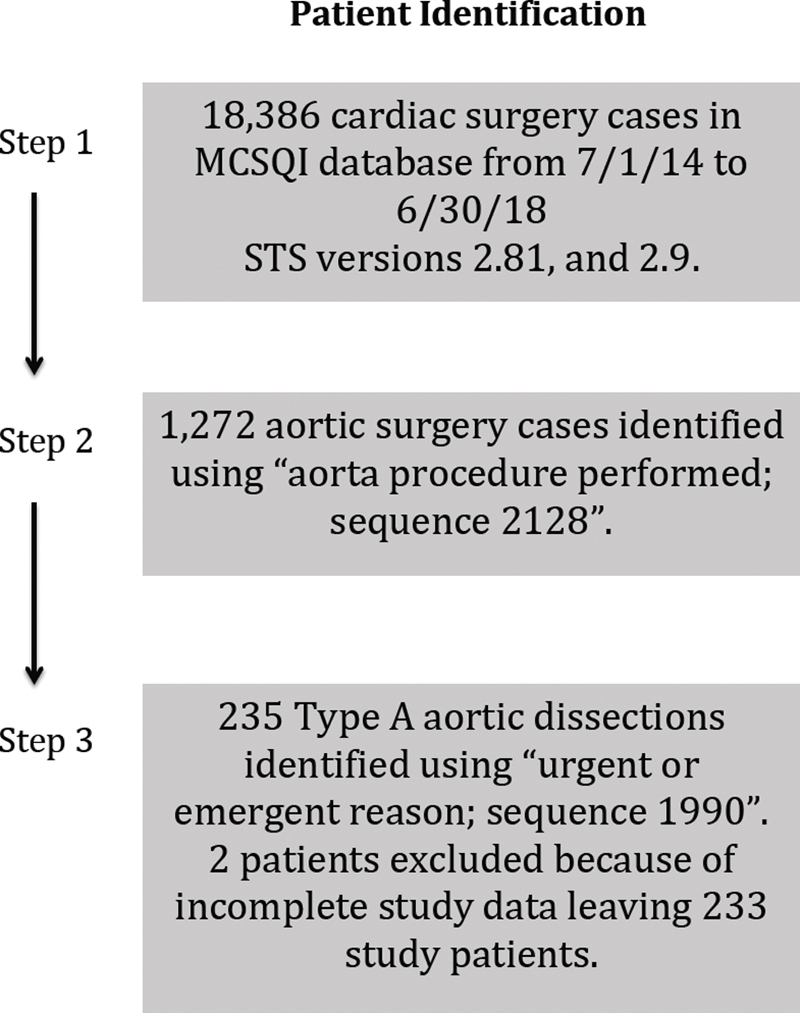
Surgery cases identified in the Maryland Cardiac Surgery Quality Initiative (MCSQI) database during the 4-year study period. STS, Society for Thoracic Surgeons.

### Study Data

For all patients, we collected the following study data: age, presence of cardiogenic shock, previous cardiac intervention, left ventricular ejection fraction (LVEF), baseline creatinine level (mg/dL), baseline hematocrit (g/dL), active tobacco use, total cardiopulmonary bypass (CPB) time, arterial cannulation site (aortic, axillary, or femoral), brain protection technique (CPB only, hypothermic circulatory arrest [HCA], HCA with antegrade selective cerebral perfusion [SCP], HCA with retrograde cerebral perfusion [RCP], or HCA with SCP and RCP), type of aortic valve operation performed, degree of baseline aortic insufficiency (AI), and the lowest body temperature during CPB. In addition to these patient level data, we collected data on total Type A aortic dissection surgery volume per hospital. All variable definitions were based on STS database definitions (versions 2.81, and 2.9).

### Study Outcomes

Study outcomes were operative mortality, blood transfusion, reoperation for bleeding, acute renal failure requiring renal replacement therapy (RRT), stroke, gastrointestinal events after surgery, total ventilation hours, and total intensive care unit (ICU) hours. Stroke etiology was described in the database as embolic, hemorrhagic, ischemic, or undetermined.

### Statistical Analysis

Statistical analysis was performed using SAS 9.3 (SAS Corporation, Cary, NC). Patient characteristics were summarized for each hospital as the median value and interquartile range or number and percentage of patients. Patient characteristics and study outcomes were compared between hospitals using the Kruskall–Wallis test (continuous variables), the Chi-squared test (categorical variables), or Fisher's exact test (categorical variables with low cell counts), as appropriate. Patients were stratified by arterial cannulation site and outcomes were compared. Patients were also stratified by brain protection technique and the overall stroke rate, as well as specific stroke types (embolic, hemorrhagic, and ischemic) were compared. The relationships between total CPB time and stroke and total HCA time and stroke were modeled using logistic regression. For all tests, *p* -values less than 0.05 were considered statistically significant and 95% confidence intervals (CIs) were calculated for all odds ratios.

## Results

A total of 18,386 cardiac surgery cases were identified in the MCSQI database during the 4-year study period ( Fig. 1 ) and a total of 1,272 aortic surgeries were performed. Eight of 10 cardiac surgery hospitals in Maryland reported 233 Stanford Type A aortic dissection repair surgeries with full study data to MCSQI. All cases except for five were done with CPB. The five cases done without CPB were with percutaneous ascending aortic endovascular stent grafts performed in a single center and all five of these patients survived to hospital discharge. The majority of patients, 158 (67.8%), were transferred from a noncardiac surgery hospital to the hospital where they had their surgery. Only seven cases were transferred from one cardiac surgery hospital to another cardiac surgery hospital for surgery. There were 28 operative deaths (12.0%) and there was no difference in mortality between patients transferred from an outside hospital or those initially admitted to the same hospital where they had their surgery (12.7 vs. 10.7%, *p* = 0.66). Six hospitals performed less than 5 cases per year on average, while two hospitals performed 10 or more cases per year on average. Table 1 lists the characteristics of patients in the eight hospitals. Patient characteristics including age, preoperative cardiogenic shock, LVEF, creatinine, and tobacco use differed significantly between patients in the eight hospitals (all *p* < 0.05). The percentage of cases done with aortic, axillary and femoral artery cannulation, and the primary brain protection technique also differed significantly between hospitals ( *p* < 0.001). Temperature management practices were significantly different with the lowest body temperature during CPB varying from 16 to 27.5°C, depending on the hospital. Finally, the percentage of surgeries with aortic valve repair or replacement procedures differed significantly between hospitals ( *p* < 0.001), although the prevalence of moderate-to-severe AI was not significantly different between ospitals ( *p* = 0.14).

**Table 1:** Patient characteristics by hospital

Variable	Hospital 1	Hospital 2	Hospital 3	Hospital 4	Hospital 5	Hospital 6	Hospital 7	Hospital 8
Type A aortic dissection surgeries	12	1	10	72	105	11	7	15
Age a	63 [58, 72]	47 [47, 47]	58 [50, 67]	58 [50, 67]	61 [54, 69]	72 [67, 78]	69 [55, 71]	52 [46, 59]
Cardiogenic shock a	1 (8.3)	0 (0.0)	0 (0.0)	8 (11.1)	0 (0.0)	3 (27.3)	0 (0.0)	2 (13.3)
Previous cardiac intervention	3 (25.0)	0 (0.0)	1 (10.0)	12 (16.7)	8 (7.6)	1 (9.1)	2 (28.6)	2 (13.3)
LVEF (%) a	55 [53, 63]	58 [58, 58]	60 [60, 60]	63 [57, 63]	55 [50, 60]	58 [53, 64]	65 [63, 65]	59 [53, 63]
Baseline creatinine (mg/dL) a	0.9 [0.7, 1.2]	0.9 [0.9, 0.9]	1.1 [1.0, 1.4]	1.2 [1.0, 1.9]	1.0 [0.8, 1.3]	1.0 [0.9, 1.4]	0.9 [0.8, 1.6]	0.9 [0.8, 1.3]
Baseline hematocrit (%)	39 [37, 42]	47 [47, 47]	38 [24, 39]	37 [32, 41]	37 [34, 41]	38 [33, 43]	39 [33, 41]	40 [38, 42]
Active smoker a	4 (33.3)	0 (0.0)	6 (60.0)	13 (18.1)	30 (28.6)	0 (0.0)	3 (42.9)	3 (20.0)
Full CPB used	12 (100.0)	1 (100.0)	10 (100.0)	72 (100.0)	100 (95.2) b	11 (100.0)	7 (100.0)	15 (100.0)
CPB time (min) a	208 [192, 238]	399 [399, 399]	179 [146, 216]	196 [160, 241]	213 [173, 255]	157 [113, 233]	144 [127, 179]	153 [135, 212]
*Arterial cannulation site* a :								
Aortic	1 (8.4)	1 (100.0)	3 (30.0)	36 (50.0)	9 (8.6)	2 (18.1)	2 (28.6)	6 (40.0)
Axillary	4 (33.3)	0 (0.0)	1 (10.0)	12 (16.7)	70 (66.7)	5 (45.5)	1 (14.3)	2 (13.3)
Femoral	7 (58.3)	0 (0.0)	6 (60.0)	24 (33.3)	21 (20.0)	4 (36.4)	4 (57.1)	7 (46.7)
*Brain protection strategy* a :								
No circulatory arrest	0 (0.0)	0 (0.0)	0 (0.0)	23 (31.9)	8 (7.6)	3 (27.3)	4 (57.1)	4 (26.7)
HCA	9 (75.0)	0 (0.0)	6 (60.0)	17 (23.6)	37 (35.2)	0 (0.0)	2 (28.5)	5 (33.3)
HCA and SCP	3 (25.0)	1 (100.0)	4 (40.0)	12 (16.7)	47 (44.8)	7 (63.6)	0 (0.0)	5 (33.3)
HCA and RCP	0 (0.0)	0 (0.0)	0 (0.0)	16 (22.2)	6 (5.7)	1 (9.1)	1 (14.4)	1 (6.7)
HCA, SCP, and RCP	0 (0.0)	0 (0.0)	0 (0.0)	4 (5.6)	7 (6.7)	0 (0.0)	0 (0.0)	0 (0.0)
Lowest temperature °C a during CPB	20.3 [19.2, 21.5]	16.0 [16.0, 16.0]	24.2 [21.2, 25.7]	27.5 [19.2, 33.8]	24.3 [20.1, 27.8]	26.0 [23.9, 33.3]	22.0 [21.5, 32.3]	20.9 [18.0, 26.6]
Moderate or severe AI c	8 (66.7)	1 (100.0)	1 (25.0)	26 (55.3)	53 (50.5)	3 (27.3)	1 (33.3)	9 (60.0)
Aortic valve replacement or repair procedure a	8 (66.7)	0 (0.0)	4 (40.0)	28 (38.9)	80 (76.2)	4 (36.4)	4 (57.1)	11 (73.3)
*Aortic valve procedure category* : a d								
AV repair or reconstruction	2 (25.0)	–	0 (0.0)	8 (28.6)	10 (12.5)	4 (100.0)	0 (0.0)	6 (54.6)
AVR	1 (12.5)	–	1 (25.0)	9 (32.1)	8 (10.0)	0 (0.0)	1 (25.0)	0 (0.0)
AVR with non-valved conduit	0 (0.0)	–	0 (0.0)	0 (0.0)	2 (2.5)	0 (0.0)	0 (0.0)	2 (18.2)
Resuspension of AV with ascending aortic replacement	5 (62.5)	–	3 (75.0)	4 (14.3)	47 (58.8)	0 (0.0)	0 (0.0)	1 (9.1)
Bentall's procedure	0 (0.0)	–	0 (0.0)	7 (25.0)	12 (15.0)	0 (0.0)	3 (75.0)	2 (18.2)
Valve sparing aortic root replacement (David's procedure)	0 (0.0)	–	0 (0.0)	0 (0.0)	1 (1.2)	0 (0.0)	0 (0.0)	0 (0.0)

Abbreviations: AI, aortic insufficiency; AV, aortic valve; AVR, aortic valve replacement; CBP, cardiopulmonary bypass; HCA, hypothermic circulatory arrest; LVEF, left ventricular ejection fraction; RCP, retrograde cerebral perfusion; SCP, selective antegrade cerebral perfusion.

a *p* -Value less than 0.05 for comparison of eight hospitals number (percentage of patients) median value and [25th, 75th percentile].

b 5 patients had ascending aortic endovascular stent grafts.

c Some patients in database missing echocardiography data, hospital 3 (6 patients), hospital 4 (25 patients), hospital 7 (4 patients).

d 1 patient from hospital 5 missing data on aortic valve procedure category.

Table 2 shows patient outcomes for the eight hospitals. Operative mortality varied between 0 and 25.0%, but was not significantly different ( *p* = 0.67) between the eight hospitals. There were two hospitals that had 0% operative mortality, but neither performed more than 10 total surgeries during the study period. The highest volume hospital had 9.5% operative mortality for 105 surgeries. Operative mortality was 11.9% for the two hospitals that performed more than 10 cases per year and 12.5% for the six hospitals that performed less than 10 cases per year. Red blood cell (RBC), fresh frozen plasma (FFP), and platelet transfusion were significantly different between the eight hospitals with the two highest volume hospitals generally transfusing more ( *p* = 0.01, 0.003, and <0.001, respectively). One low-volume hospital that performed 15 total cases during the study period had no reoperations for bleeding and a very low transfusion rate. There were no differences in clinical outcomes including reoperations for bleeding, acute renal failure requiring RRT, stroke, gastrointestinal complications, total ventilation hours, and total ICU hours between the eight hospitals (all *p* > 0.05).

**Table 2:** Patient outcomes by hospital

Variable	Hospital 1	Hospital 2	Hospital 3	Hospital 4	Hospital 5	Hospital 6	Hospital 7	Hospital 8
Operative mortality	3 (25.0)	0 (0.0)	0 (0.0)	11 (15.3)	10 (9.5)	1 (9.1)	1 (14.3)	2 (13.3)
RBCs during surgery (units) a	0 [0, 3]	0 [0, 0]	4 [2, 4]	3 [0, 8]	3 [1, 6]	2 [2, 5]	1 [0, 2]	0 [0, 4]
Platelets during surgery (units) a	4 [4, 6]	3 [3, 3]	4 [4, 7]	2 [1, 3]	3 [2, 3]	2 [0, 4]	2 [1, 2]	2 [1, 4]
FFP during surgery (units) a	3 [2, 6]	4 [4, 4]	5 [4, 8]	4 [2, 11]	5 [3, 7]	4 [0, 6]	2 [1, 2]	2 [1, 4]
Reoperation for bleeding	2 (16.7)	0 (0.0)	0 (0.0)	12 (16.7)	4 (3.8)	1 (9.1)	0 (0.0)	0 (0.0)
Renal failure requiring RRT	1 (8.3)	0 (0.0)	0 (0.0)	14 (19.4)	11 (10.5)	2 (18.2)	0 (0.0)	1 (6.7)
Stroke	0 (0.0)	0 (0.0)	1 (10.0)	10 (13.9)	11 (10.5)	0 (0.0)	1 (14.3)	2 (13.3)
GI event	1 (8.3)	0 (0.0)	0 (0.0)	3 (4.2)	8 (7.6)	3 (27.3)	0 (0.0)	2 (13.3)
Total ventilation hours	13 [8, 112]	11 [11, 11]	64 [31, 90]	39 [8, 133]	19 [7, 63]	26 [10, 73]	22 [15, 40]	21 [7, 31]
Total ICU hours	71 [46, 200]	112 [112, 112]	118 [72, 152]	135 [54, 316]	105 [60, 204]	74 [25, 104]	167 [96, 216]	59 [27, 94]

Abbreviations: FFP, fresh frozen plasma; GI, gastrointestinal; ICU, intensive care unit; RBC, red blood cell; RRT, renal replacement therapy.

a *p* -Value less than 0.05 number (percentage of patients) median value and [25th, 75th percentile].

Table 3 shows clinical outcomes after stratification by arterial cannulation site. Operative mortality did not differ significantly after stratification by arterial cannulation site: 6.7% for patients who had aortic cannulation ( *n* = 60), 17.8% for patients who had femoral artery cannulation ( *n* = 73), and 11.6% for patients who had axillary artery cannulation ( *n* = 95; *p* = 0.14). There were no statistically significant differences in rates of acute renal failure requiring RRT, stroke, or gastrointestinal events between patients with different arterial cannulation sites (all *p* > 0.05).

**Table 3:** Outcomes stratified by arterial cannulation site

Variable	Aorta ( *n* = 60)	Femoral artery ( *n* = 73)	Axillary artery ( *n* = 95)	*p* -Value
Cardiogenic shock	6 (10.0)	3 (4.1)	5 (5.3)	0.46
CPB time	182 [136, 239]	207 [174, 246]	201 [169, 245]	**0.04**
Operative mortality	4 (6.7)	13 (17.8)	11 (11.6)	0.14
Renal failure requiring RRT	12 (20.0)	8 (11.0)	9 (9.5)	0.14
Stroke	9 (15.0)	8 (11.0)	8 (8.4)	0.44
GI event	3 (5.0)	8 (8.4)	6 (8.2)	0.70

Abbreviations: CPB, cardiopulmonary bypass; GI, gastrointestinal; RRT, renal replacement therapy.

Table 4 shows stroke rates after stratification by brain protection technique. The overall stroke rate for the cohort was 10.7% and embolic strokes were most frequent. Patients who had CPB without HCA had a stroke rate of 11.9%, patients who had HCA without adjunct brain perfusion had a stroke rate of 10.5%, patients who had HCA with SCP had a stroke rate of 10.1%, patients who had HCA with RCP had a stroke rate of 8.0%, and patients who had HCA with SCP and RCP had a stroke rate of 20% ( *p* = 0.92). Of note, the embolic stroke rate was 9.5% in 42 patients who did not have HCA and had aortic cross-clamping or endovascular repair, while it was 1.6% in 191 patients who had HCA with or without adjunct brain perfusion ( *p* = 0.03). There was no significant relationship between total CPB time and stroke on univariate logistic regression analysis, meaning that total CPB time was not associated with stroke in our analysis. The odds ratio for stroke was 0.998 (95% CI: 0.992–1.005, *p* = 0.61) for each CPB minute. For patients who had HCA, with or without adjunct brain perfusion, there was no significant relationship between total HCA time and stroke; odds ratio for stroke was 1.004 (95% CI: 0.970–1.039, *p* = 0.82) for each HCA minute. Median total HCA time without brain perfusion was 5 minutes [0, 19] for patients in the cohort. Of note, the two highest volume hospitals in the state used different brain protection techniques when HCA was required. The highest volume hospital used HCA with SCP in 44.8% of its surgeries, while the second highest volume hospital used HCA with SCP in only 16.7% of its surgeries.

**Table 4:** Strokes stratified by brain protection strategy

Variable	No HCA a ( *n* = 42)	HCA ( *n* = 76)	HCA + SCP ( *n* = 79)	HCA + RCP ( *n* = 25)	HCA + SCP + RCP ( *n* = 11)	*p* -Value
Permanent stroke	5 (11.9)	8 (10.5)	8 (10.1)	2 (8.0)	2 (18.2)	0.92
*Stroke etiology* :						
Embolic	4 (80.0)	1 (12.5)	0 (0.0)	0 (0.0)	2 (100.0)	0.06
Hemorrhagic	0 (0.0)	2 (25.0)	2 (25.0)	1 (50.0)	0 (0.0)	
Ischemic stroke	0 (0.0)	1 (12.5)	3 (37.5)	1 (50.0)	0 (0.0)	
Undetermined	1 (20.0)	4 (50.0)	3 (37.5)	0 (0.0)	0 (0.0)	

Abbreviations: HCA, hypothermic circulatory arrest; RCP, retrograde cerebral perfusion; SCP, antegrade selective cerebral perfusion.

a Patients who did not have HCA had more embolic strokes than those who had HCA with or without adjunct brain perfusion ( *p* = 0.03).

## Discussion

In our study, 8 of 10 cardiac surgery hospitals in Maryland reported 233 Stanford Type A aortic dissection repair surgeries to MCSQI over a 4-year period. To our knowledge, our study is one of the first to examine statewide practice variation for acute aortic dissection repair surgery. Our study's major findings with regard to Stanford Type A aortic dissection repair surgery in Maryland were (1) operative mortality was similar for high and low volume hospitals; (2) there were significant differences in patient comorbidities, operative techniques, and blood transfusion between hospitals; (3) arterial cannulation sites were variable but did not have a significant association with outcome; (4) embolic strokes were the most common type of stroke; (5) aortic cross clamping without HCA was associated with an increased risk of embolic stroke; and (6) HCA brain protection strategies were variable but had no significant association with stroke.

Stanford Type A aortic dissection has a high mortality rate without timely surgical correction. The primary goal in patients is to normalize blood flow to the true aortic lumen so that perfusion is restored to the heart, kidneys, intestinal organs, and extremities and to replace the ascending aorta to prevent cardiac tamponade or rupture. An experienced and readily available team is needed, which includes a cardiothoracic surgeon, vascular surgeon, general surgeon, perfusionist, and cardiothoracic anesthesiologist. In some regions of the United States, Stanford Type A aortic dissection repair surgery is performed primarily in tertiary care hospitals, whereas in other regions, timely transport to a tertiary care hospital is not feasible and cases must be done in smaller regional hospitals. There is a clear risk-benefit balance that must be considered by cardiothoracic surgeons in smaller regional hospitals. For each hour without surgical correction, the mortality rate increases by 1 to 2%. 2 However, operative mortality may be higher in smaller hospitals, particularly if supportive resources are not readily available (e.g., vascular surgeon, 24-hour physician critical care staffing, blood bank resources, and so on). Further, some advanced techniques may not be available in smaller regional hospitals, and it is unclear whether lower volume hospitals have adopted more contemporary techniques for arterial cannulation and brain protection.

Case volumes are well known to affect outcomes in complex cardiac surgery procedures. For example, in complex mitral valve repair surgery individual surgeon volume strongly predicts successful mitral valve repair and freedom from reoperation. 5 Similarly, hospital volume is strongly associated with survival after the placement of left ventricular assist device (LVAD). 6 Emergency surgical procedures have a disproportionately high risk for mortality and an important question is whether centers with different emergency surgery volumes have equivalent outcomes. The published literature is not definitive on this topic. In one study in Maryland, high-volume emergency general surgery hospitals had better outcomes than low-volume emergency general surgery hospitals. 7 The same group of authors showed that in geriatric patients the effect size was even greater with low volume emergency surgery hospitals having an 86% higher risk for death. 8 Alternatively, in a report published by the Nuffield Trust and commissioned by the Royal College of Surgeons, high volume emergency surgery units did not have better outcomes than low volume units. 9 These dramatically different findings suggest that the relationship between emergency surgery volume and outcome may differ depending on the specific geographic location and health care system.

There are limited data about Stanford Type A aortic dissection repair surgery volume and outcome. In one study that included over 5,000 acute aortic dissection patients, surgical volume was strongly correlated with patient outcome. 4 In a second analysis performed in the United Kingdom, which included data from 249 cardiothoracic surgeons, acute aortic dissection volume was similarly associated with patient outcome. 10 In this study, performing an average of five or more thoracic aortic replacement surgeries per year was associated with better outcomes. 10 To our knowledge, our study represents one of the first detailed statewide analyses of Stanford Type A aortic dissection repair surgery in the United States. In our study, operative mortality was similar among eight Maryland hospitals and higher surgical volume was not associated with superior outcome.

Based on our analysis, it appears that Stanford Type A aortic dissection repair surgery can be safely performed in select regional cardiac surgery hospitals within Maryland. In a recent review of Stanford Type A aortic dissection repair outcomes within the United States, operative mortality was 17% and in Maryland operative mortality was 12%, which compares favorably. 3 This finding has important ramifications for the triage of patients within our state. Although the majority of critically ill patients can be safely transported between hospitals, 11 12 it appears that well-selected patients with Stanford Type A aortic dissection can have surgical repair with comparable outcomes in smaller regional cardiac surgery hospitals, where surgeons have expertise in aortic surgery. Patients can be transported either by helicopter or ambulance to a tertiary care hospital in Maryland but sometimes weather conditions preclude a timely transfer. In addition, there is significant cost associated with interhospital transfer and the time required for transfer can negatively impact patient outcome.

Nevertheless, we believe that some complex Stanford Type A dissection cases may be best handled in a tertiary care hospital and that development of “centers of excellence” probably allows for high-risk patients to achieve better than expected outcomes. For example, ascending aortic endovascular stent grafts have been used in select high-risk patients with Stanford Type A aortic dissection and five such cases were done at the highest volume hospital during the 4-year study period. 13 14 15 All five patients survived to hospital discharge and there were no strokes observed. For these cases, percutaneous common femoral arterial access is obtained and a 10-cm endovascular stent graft is brought retrograde via the descending thoracic aorta into the proximal ascending aorta near the intimal tear. The endograft is deployed during a brief period of rapid ventricular pacing. This type of approach has been shown to have comparable short-term mortality when compared with open repair and a low rate of endoleak. 15

Another important aspect of our study is that we identified potentially modifiable surgical factors that were variable between hospitals and may offer an opportunity for greater standardization and process improvement in Maryland. As an example, temperature management during HCA differed significantly between hospitals with nadir temperature varying from 16 to 27°C depending on the hospital. In the two highest volume hospitals, nadir temperature was kept in the moderate range (24–27°C), while in several lower volume hospitals, deeper hypothermia (20–22°C) was used. Temperature management during HCA could have a significant impact on patient outcome because deep hypothermia is associated with more bleeding and moderate hypothermia has been shown to have comparable neurologic outcomes in contemporary studies. 16 17 Arterial cannulation site and brain protection technique also differed significantly between hospitals, which is not surprising and in some cases may have been mandated by surgical pathology or other clinical conditions. The optimal arterial cannulation site and brain protection technique for thoracic aortic replacement surgery remain controversial and even in recent expert consensus guidelines, there was no strong recommendation for a specific cannulation site or brain protection technique. 18 19 In fact, a 2010 multisociety guideline stated that “institutional experience is an important factor in selecting these techniques.” 19 In a recently published study of over 7,000 Stanford Type A aortic dissection surgeries, using data from the national STS database, femoral arterial cannulation was associated with a higher risk of stroke, while HCA with RCP was associated with a reduced risk of stroke. 20 These findings are consistent with our own study data, as patients who had femoral artery cannulation had the highest operative mortality in our study (17.8%) and patients who had HCA with RCP had the lowest stroke rate (8%).

## Limitations

Our study has several important limitations. First, it is retrospective and we cannot rule out the possibility of unobserved confounding. Second, some aortic dissection cases were likely missed because of misclassification, missing data, improper coding, or because the case was not abstracted to MCSQI from the contributing data center. Third, some strokes were probably subclinical and were missed by our analysis. Similarly, some strokes may have been present preoperatively but were identified postoperatively. Fourth, it is difficult to compare the severity of illness of patients because we did not have data on preoperative lactate levels or degree of shock. Along these lines, the STS database does not have a specific risk score for Stanford Type A aortic dissection patients. Fifth, our study cannot account for whether patients may have been turned down for surgery at different hospitals and, hence, there could be selection bias. Sixth, we did not have granular detail on aortic dissection anatomy, extent, and whether visceral malperfusion occurred before surgery. It is possible that different hospitals excluded patients with different pathology leading to bias. Finally, our study was underpowered to detect small differences in outcomes between hospitals and so negative findings cannot be considered definitive.

## Conclusion

In conclusion, our analysis suggests that well selected patients can have Stanford Type A aortic dissection repair surgery safely performed with comparable outcomes at smaller regional cardiac surgery hospitals within Maryland. We also found that hospitals within the state had significant clinical practice variation in terms of modifiable surgical variables, which may offer an opportunity for greater standardization and process improvement in the future. These findings have important implications for triage and management of acute aortic dissection patients both within Maryland and nationally.

### Editor’s Questions

Is it possible that the better results with axillary cannulation over femoral or direct aortic cannulation are related to patient acuity? In other words, is it possible that the expediency of femoral or direct aortic cannulation (compared to the more time consuming axillary exposure) was chosen in sicker patients–perhaps those in shock or with active end organ ischemia. Higher patient acuity could thus account for the survival benefit of axillary cannulation. Please comment.

*The prevalence of cardiogenic shock at the time of presentation was not significantly different between axillary, femoral and direct aortic cannulation (5.3, 4.1, and 10.0%). Recent studies have shown that arterial cannulation strategy impacts the incidence of adverse postoperative neurologic outcomes; axillary cannulation with the lowest risk of postoperative stroke and femoral artery cannulation with the highest risk of stroke. 20 Our study findings are notable in that they demonstrate that direct aortic cannulation with cross clamping is associated with a significantly higher rate of embolic stroke than using hypothermic circulatory arrest. Despite a higher embolic stroke rate, patients who had aortic cannulation actually had the lowest operative mortality (6.7%) in our study.*

*The optimal arterial cannulation site remains an individual patient decision in our opinion, but we do feel that the preponderance of evidence from multiple studies suggests that femoral artery cannulation may not be ideal for acute aortic dissection repair surgery. This being said, in cases where the patient is arresting on arrival to the operating room, there may be no other choice for rapid cannulation to initiate cardiopulmonary bypass. For cases where there is more time to select an arterial cannulation site, we feel a randomized trial to investigate the impact of cannulation site on postoperative mortality seems necessary.*
